# Swiss national radon database: impact of building and environmental factors

**DOI:** 10.3389/fpubh.2025.1625922

**Published:** 2025-08-22

**Authors:** Joan F. Rey, Caterina Berlusconi, Luca Pampuri, Joëlle Goyette Pernot

**Affiliations:** ^1^Western Switzerland Center for Indoor Air Quality and Radon (croqAIR), Transform Institute, School of Engineering and Architecture of Fribourg, HES-SO University of Applied Sciences and Arts Western Switzerland, Fribourg, Switzerland; ^2^Department of Environment Constructions and Design (DACD), Radon Competence Centre (CCR), University of Applied Sciences and Arts of Southern Switzerland (SUPSI), Manno, Switzerland

**Keywords:** radon database, radon exposure, environmental influences, geogenic potential, climate influences, building characteristics, public health

## Abstract

Since the 1980s, radon has been recognized as a public health concern in Switzerland and internationally. In an effort to more accurately estimate the number of lung cancer cases attributable to radon exposure, Swiss health authorities initiated the creation of radon measurements into a centralized national database. As of 2025, this database comprises approximately 300,000 measurements from 150,000 buildings across the country. This study aims (1) to provide a statistical characterization of the Swiss National Radon Database, including temporal and structural aspects (e.g., number of measurements, measurement duration), and (2) to identify key environmental and anthropogenic factors influencing indoor radon concentrations, using various national datasets (e.g., geology, hydrology, climate, seismicity, soil chemistry, building characteristics). Results indicate that elevated indoor radon levels are primarily associated with the presence of uranium-rich geological formations and fault zones, particularly within karstic environments. Among building-related parameters, older constructions and lower floor levels are linked to higher radon concentrations, while building type appears to have minimal influence. Moreover, a negative correlation was observed between measurement duration and radon levels, suggesting that shorter measurements tend to overestimate long-term exposure and raising questions regarding the annual representativeness. This study provides a comprehensive overview of radon distribution patterns and their determinants, offering valuable insights for researchers and public health authorities. It contributes to the development of evidence-based strategies for radon risk assessment, management, and mitigation, both within Switzerland and in comparable international contexts.

## 1 Introduction

Radon is a naturally occurring radioactive noble gas that originates from the decay of uranium in soil, rocks, and water (1). Being colorless, odorless, and tasteless, radon can accumulate in enclosed spaces, such as buildings, and pose significant health risks (1–3). Long-term exposure to radon is a well-documented cause of lung cancer, making it a public health concern worldwide. Moreover, the combined exposure to radon and smoking significantly amplifies the risk of developing lung cancer (1, 4–6). Radon concentrations vary geographically, depending on different factors, such as geological formations, soil properties, building characteristics and occupants behaviors (1, 3, 7).

Since the 1980s, Switzerland recognized the importance of monitoring radon levels due to its diverse geological landscape, which includes uranium-rich granite regions (2, 8). Several factors strongly influence radon distribution across the country, including geological formations, soil permeability, building materials, and ventilation practices (7, 9–14). Additionally, seasonal variations, meteorological and climatic conditions contribute to fluctuations in indoor radon levels (7, 15). The Swiss government has implemented various policies to regulate radon exposure, including the RPO (Radiological Protection Ordinance) revised in 2017, and based on RPA (Radiological Protection Act) of 1991 (16, 17). Two national action plans were issued from this legislation (4, 18). The Radon action plan 2021–2030 currently covers four different lines of action toward radon mitigation: building stock; health risk; radon expertise; and employee protection (4).

Radon measurements in Switzerland have been collected since 1981 through extensive monitoring programs, initiated by national, cantonal and municipal authorities, and also by individual initiatives. To be included in this database, measurements must meet the criteria for official measurements. Since 2018 and the application of the latest version of the RPO (16), an official measurement has been defined as a passive year-long assessment or, at a minimum, a three-month measurement during the heating season (October to March) (19). However, this definition has evolved since the 1980s, and shorter 1-month-measurement durations were acknowledged as official until the end of 2017.

Data are centralized and stored by Switzerland's Federal Office of Public Health (FOPH), who has developed a federal radon database (20) that, as of the end of 2020, contains ~300,000 indoor radon measurements collected from 150,000 buildings. This database is one of the largest and densest in the world (21, 22). In fact, the Swiss radon database has a density of 3.5 buildings investigated for radon per square kilometer. This represents approximately at least a radon measurement carried out in 6 out of 100 buildings across the country (4, 23). This dataset is a valuable resource for various purposes, including scientific research, policy development, and targeted public information campaigns (4, 12, 13, 21, 24).

Several countries have developed national radon databases, such as USA, France, Italy, Germany and Austria to support research and public health initiatives (25). However, the absence of common standards (e.g., measure, unit) complicates cross-comparison between databases (26).

Despite the size and content of the database, significant gaps remain regarding trends and relationships among the included variables. This study addresses the following research questions: (1) Which variables are most consistently reported across the database? (2) Do relationships exist between recorded variables and high radon concentrations? (3) Based on the findings, what measures can relevant authorities implement to improve radon exposure and management in Switzerland? We hypothesize that a comprehensive analysis of the existing dataset is crucial for understanding the specific characteristics of radon related issues in Switzerland. Addressing this topic is necessary to develop the most targeted mitigation solutions and prevention measures, according to different geographical specificities (i.e., urban context, topographic context) which strongly influences building typologies and occupants' behaviors. Therefore, this paper aims at (1) statistically describe the Swiss national radon database (duration, building type), highlighting the different variables and their relative specificity, and (2) according to different Swiss datasets available (i.e., geology, hydrology, climate, seismic area, soil chemistry), identify key factors influencing radon distribution in Switzerland.

## 2 Methodology

### 2.1 The Swiss federal database

Following an authorization process, the radon database was obtained from the Swiss Federal Office of Public Health. The dataset, provided as an Excel file, compiles all official radon measurements conducted across various locations up to March 5, 2025. Descriptive statistics of the initial dataset are provided in the first section of the results.

### 2.2 Datasets collection

Datasets were collected from multiple sources. Table 1 provides a summary of the datasets and presents relevant information.

**Table 1:** Dataset collected for statistical analyses.

**Theme**	**Source**	**Data type**	**Availability**	**Version**	**Extent and resolution**	**Reference**
Radon	FOPH^a^	Database	On request	March 2025	Not applicable	Not applicable
Geology	Swisstopo	Vector	Opensource	June 2014	Switzerland, 1:500,000	(66)
Uranium	FOEN^b^	Raster	Opensource	December 2023	Switzerland, 1 x 1 km	(34, 35)
Seismic risk zone	FOEN^b^	Vector	Opensource	March 2011	Switzerland	(36)
Climatological norm 1991–2020^c^	MeteoSwiss	Raster	Opensource	2021	Switzerland, 1 x 1 km	(67)

^*a*^FOPH, Federal Office of Public Health; ^*b*^FOEN, Federal Office of Environment; ^*c*^Climatological 1991–2020 norms for daily average temperature and average precipitation sum were retrieved.

### 2.3 Merging the datasets

The retrieved datasets originate from various sources and encompass a wide range of formats, including databases, vector layers and raster images. All data were imported into R software to facilitate a unified analysis within a single platform. The analyses were conducted using the sf (27, 28) and terra (29) packages in R (version 4.3.2−2023-10-31). Graphical representations were produced using ggplot2 package (30).

### 2.4 Statistical analysis

The Wilcoxon–Mann–Whitney test (31) was used to assess statistical differences between the distributions. A *p*-value threshold of 0.05 was considered for statistical significance.

## 3 Results

### 3.1 Generalities

First, we investigated the completeness of the different variables. This step was realized on the database without any modifications. On March 5, 2025, the Swiss national radon database contained 26,948 radon measurements coming from 151,956 buildings.

Among the 41 variables, 20 were fully completed, including canton, building ID, address, Municipality, building category, measure protocol and ID, measurement instrument type and number, investigated space type and ID, occupancy, floor and finally, remediation deadline. Similarly, some variables reach high completion scores (>90% of completion) such as beginning and end of measurements, radon levels and uncertainty (cf. Table 2 and Supplementary Table S1). Geographical coordinates of measurements were completed in 97% of the time, which allows us and other researchers to spatially analyze the different data. Finally, it has to be noted that the EGID (Federal Building Identification Number), introduced in 2008, is only presented 70% of the time. This may represent a challenge when overlapping the dataset with different databases, such as the FOS register of building.

**Table 2:** Database variable completion score.

**Less answered variables**		**Most completed variables**	
**Variable**	**% of completion**	**Variable**	**% of completion**
Foundation structure	16.77%	20 variables were completed^a^	100%
Ventilation system	17.37%	Locality	99.79%
Number of underground floors	17.77%	Measure start	99.96%
Slope	18.02%	Measure end	99.76%
Building number of floors	24.16%	Radon level	99.54%

The full table is presented in Supplementary Table S1 along with variable descriptions.

^*a*^20 variables list: canton, building ID, address, postal number, municipality ID, municipality, building category, geo coordinate source, ID measurement, measurement protocol, measure type, measurement instrument ID, measurement instrument type, space ID, space type, occupancy, floor, mitigation deadline (if applicable), detection method, floor category.

**Figure 1:**
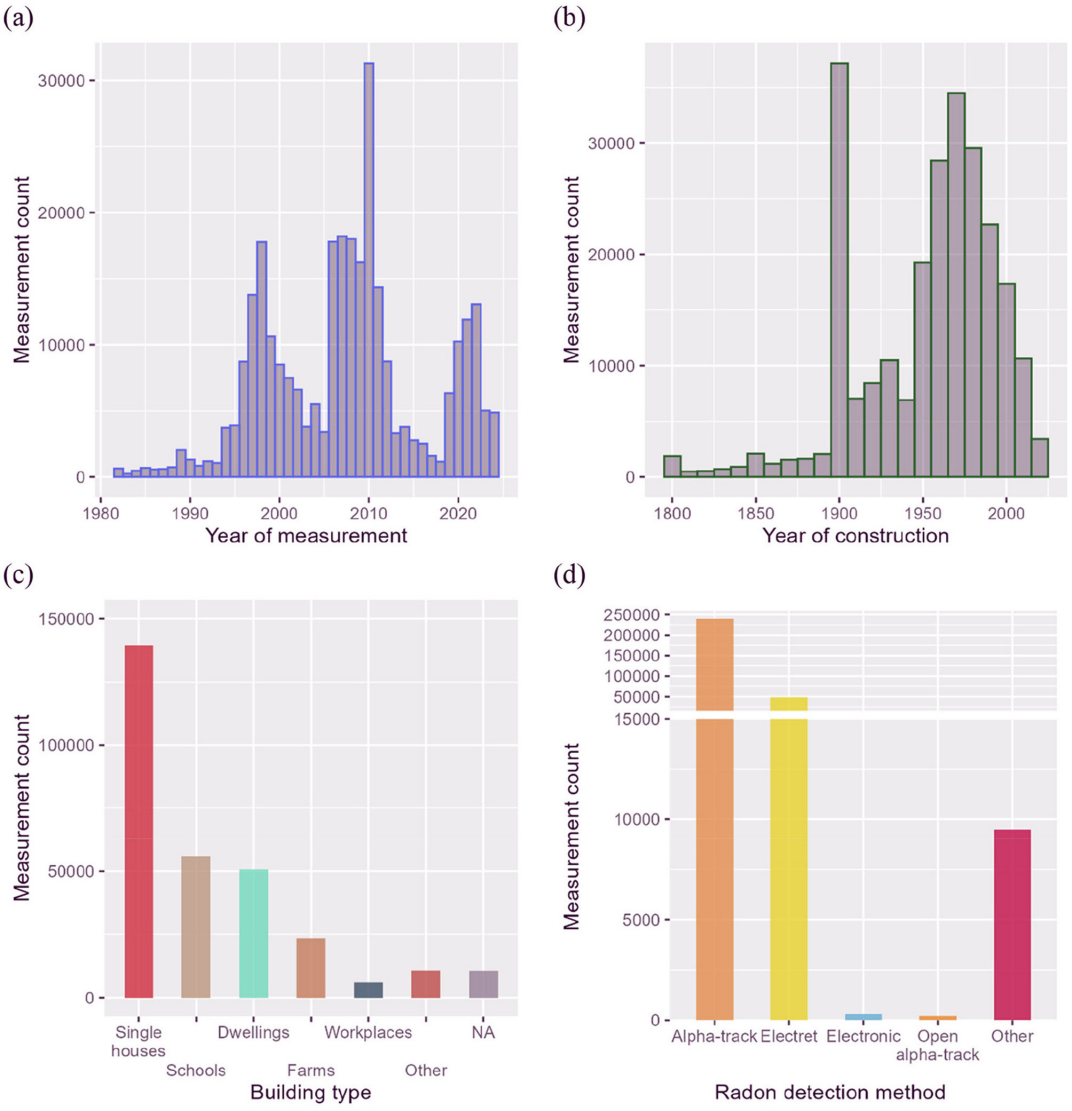
General overview of the Swiss radon database. Histograms of the number of radon measurements according to **(a)** year of measurement, **(b)** the age of the buildings, **(c)** the building category, and **(d)** the detection method.

Figure 1 presents radon measurements categorized according to various features. Initially, the number of measurements varied significantly over time. Three distinct periods, highlighted in Figure 1a as peaks in 1998, 2010, and 2022. 1998′s peak corresponds to the maximum number of national campaigns deployed with the help of the cantons for the implementation of the radon cadaster. 2010′s peak follows the publication by the WHO and the WHO's 2010 recommendation not to exceed 300 Bq/m^3^. Since 2019, continuous growth has been observed, linked to the revision of the ordinance coming into effect, along with the impact of SARS-CoV-2 between 2020 and 2022. The age distribution of the investigated buildings shows a peak in the 1970s (Figure 1b). The peak recorded for the decade 1900′s is due to automatic attribution of the value 1900 when the actual construction date was unknown for buildings built between 1900 and 1940. The majority of the surveyed buildings were single houses (139,458 measurements in 86,810 buildings), followed by schools (56,113 measurements in 12,227 buildings) and residential dwellings (50,792 measurements in 29,139 buildings). Office buildings were proportionally less investigated (5,907 measurements in 2,143 buildings). Other building types include water supply installations, tourist caves, research facilities, thermal baths, mines, food cellars, wine cellars, and parking lots (Figure 1c). Finally, most measurements were conducted using the alpha-track detection method, followed by electret-based techniques (Figure 1d). Other measurement approaches include unspecified methods and non-attributed techniques.

### 3.2 Distributions of radon levels

**Figure 2:**
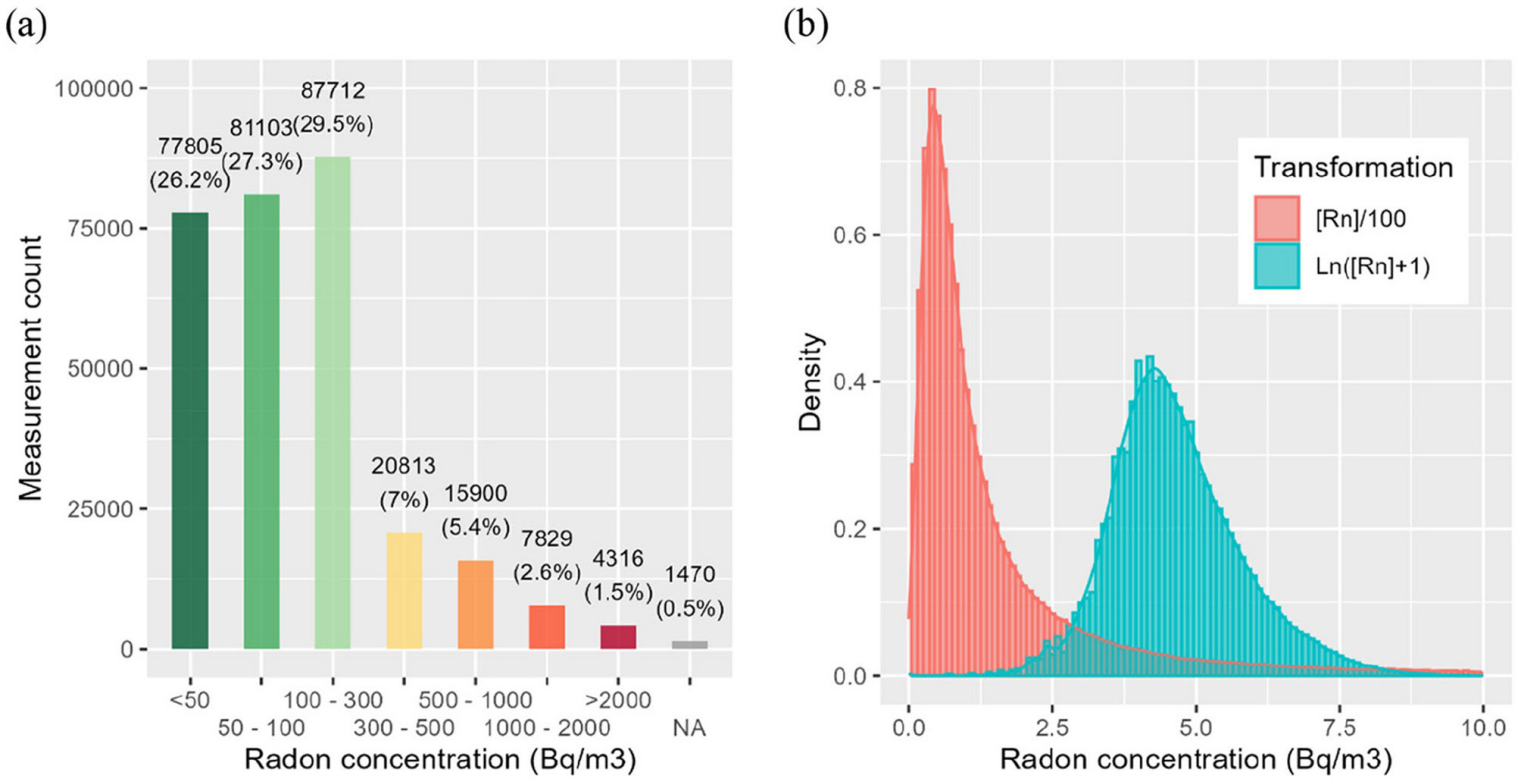
**(a)** Absolute radon concentrations distributed according to defined ranges and **(b)** density curves of absolute (scaled) and log normalized radon concentrations distributions.

Figure 2a presents several key elements. In Switzerland, most of measured radon levels were below 300 Bq/m^3^. This distribution is supported by statistical analysis, with the median and mean values recorded at 91 Bq/m^3^ and 237 Bq/m^3^, respectively, indicating that exceptionally high values skew the mean. Notably, 83.45% of the distribution falls below 300 Bq/m^3^. The 16.55% of measurements exceeding the reference values correspond to 48,858 measurements conducted in 30,390 different buildings. This corresponds to a total of 17,341 single houses, 5,737 dwellings, 2,346 farms, 2,338 schools, 489 workplaces and some buildings defined as other. Table 3 presents in detail the descriptive statistics of radon levels measured in Switzerland.

**Table 3:** Descriptive statistics of radon concentrations in Switzerland and the number of measurements and buildings exceeding threshold values.

**Descriptive statistics**	**Value**	**Measurements over the threshold value**	**Buildings over the threshold value**
No. of measurements	296,948	–	–
Buildings investigated	151,956	–	–
Mean	237 Bq/m^3^	62,424	38,999
Geometric mean	105 Bq/m^3^	131,439	80,212
Standard deviation	632 Bq/m^3^	-	-
Percentile 1	9 Bq/m^3^	292,250	151,341
Percentile 5	20 Bq/m^3^	279,942	148,406
Percentile 10	29 Bq/m^3^	264,076	143,185
Percentile 25	49 Bq/m^3^	219,928	124,950
Median	91 Bq/m^3^	147,339	88,965
Percentile 53.5	100 Bq/m^3^	136,570	83,087
Percentile 75	200 Bq/m^3^	73,652	45,978
Percentile 83,45	300 Bq/m^3^	48,858	30,390
Percentile 90	477 Bq/m^3^	29,531	18,173
Percentile 92,31	600 Bq/m^3^	22,699	13,934
Percentile 95	858 Bq/m^3^	14,771	8,964
Percentile 95.89	1,000 Bq/m^3^	12,145	7,359
Percentile 99	2,518 Bq/m^3^	2,956	1,919
Max	39,100 Bq/m^3^	-	-

Figure 2b illustrates the density of radon concentrations measured in Switzerland using two transformations. First, absolute radon concentrations [Rn] were divided by 100, resulting in a distribution that follows a Poisson distribution pattern. Second, radon concentrations were log-transformed (natural log) after adding 1 to avoid negative infinity values. Following this log-normal transformation, the distribution approximates normality visually, but statistical tests for normality (e.g., Kolmogorov-Smirnov: *D* = 0.055, *p*-value < 2.2 × 10^−16^; Anderson-Darling: *A* = 1,750.5, *p*-value < 2.2 × 10^−16^ ) reject the null hypothesis, which underline a significant deviation from a Gaussian distribution. This difference may be explained by the very large sample size, which makes even the slightest difference statistically significant. Therefore, throughout the entire study, the statistical tests used are non-parametric since radon data do not follow a normal distribution.

### 3.3 Impact of measurement duration

**Figure 3:**
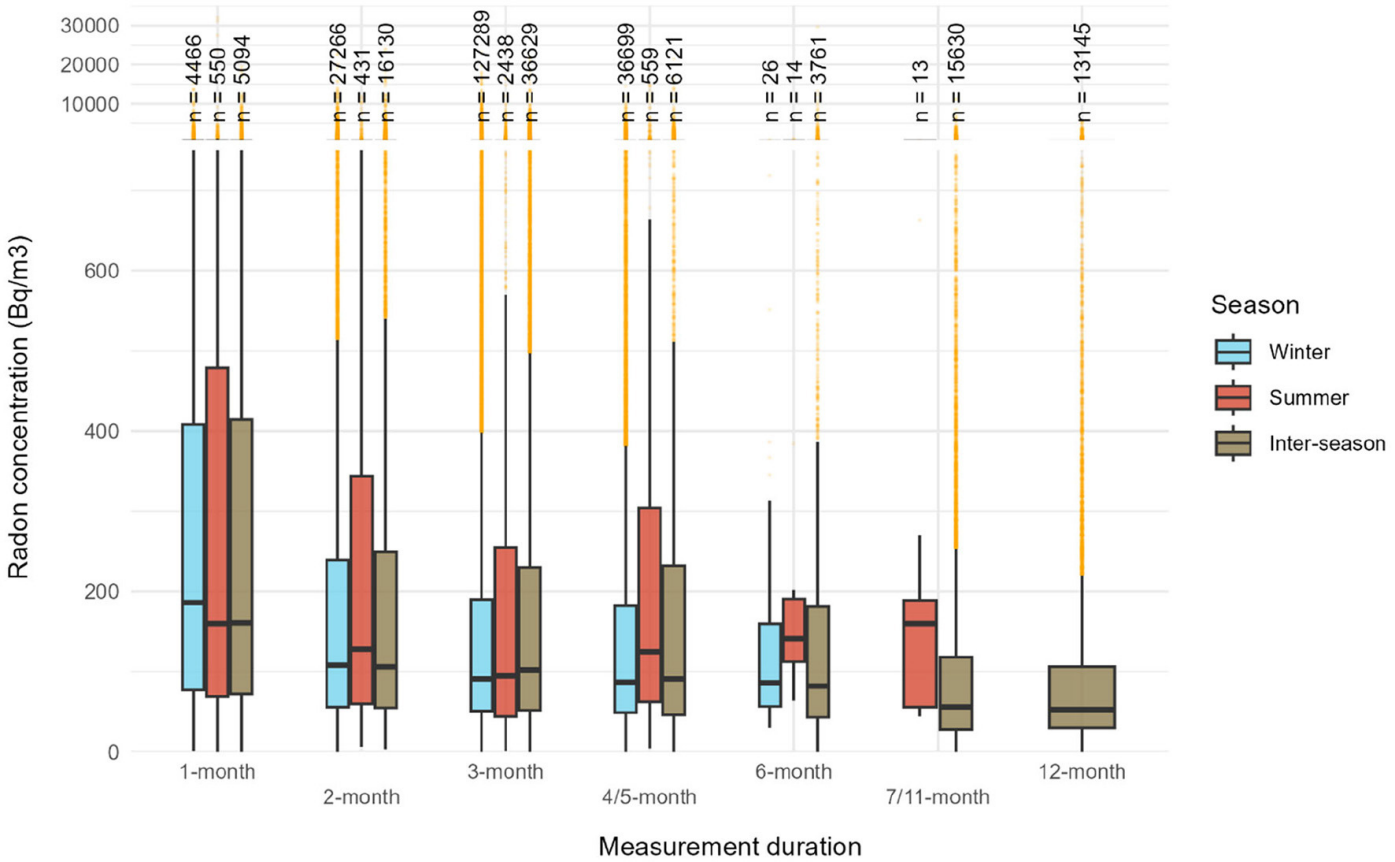
Radon concentrations distributed according to measurement length (1-month: < 59 days; 2-months: 59–88 days; 3-months: 89–118 days; 4/5-months: 119–179 days; 6-months: 180–210; 7/11-months: 211–364; 12-months: >364 days) and the season when the measurement mainly took place (winter refers to the months of December, January and February, summer to June, July and August, and inter-season to the rest of the year).

Figure 3 presents radon concentration distributions based on measurement duration and the season when the measurement mainly took place. Figure 3 depicts several elements. At first, we observed that medians for measurements carried out mostly during winter and inter-season decreased with longer measurement periods. This observation is also valid for the 75th percentile. The median concentration across all seasons decreased from 170 to 53 Bq/m^3^ for 1-month and 12-month measurements, respectively, while the 75th percentile dropped from 400 to 100 Bq/m^3^.

Secondly, Table 4 reports the p-values from Wilcoxon-Mann-Whitney and Kolmogorov-Smirnov tests, highlighting no significant trend, all durations included. No statistically significant differences were observed between the 3-months summer and winter distributions according to Wilcoxon–Mann–Whitney tests (Table 4) highlighting that 3-month measurements conducted during winter and summer were overall similar, despite that seasonal correction factors were not used. This similarity persists even though, in Switzerland, only measurements taken during the heating period are considered official. To support this finding, we observed that median radon concentrations were 91 Bq/m3 in winter and 95 Bq/m3 in summer, with corresponding 75th percentiles of 190 and 255 Bq/m3, respectively. Supplementary Figure S1 provides radon concentrations distributions according to measurement length and regardless of the season.

**Table 4:** *p*-values of Wilcoxon–Mann–Whitney (WMW) and Kolmogorov-Smirnov (KS) tests applied to compare distributions.

**Durations**	**Distributions** ^**a**^		
	**Winter vs. summer**	**Winter vs. inter-season**	**summer vs. inter-season**
1-month	WMW: 0.8809 KS: 0.0094^**^	WMW: 0.0241 KS: 0.0001^***^	WMW: 0.4415 KS: 0.0094^**^
2-month	WMW: 0.0011^**^ KS: 0.0005^***^	WMW: 0.4634 KS: 0.0908	WMW: 0.0008^***^ KS: 0.0024^**^
3-month	WMW: 0.0501 KS: 4.4 × 10^−11^***	WMW: < 2.2 × 10^−16^*** KS: < 2.2 × 10^−^16***	WMW: 0.1551 KS: 3.36 × 10^−6^^***^
4/5-month	WMW: 1.376 × 10^−14^^***^ KS: 6.55 × 10^−12^^***^	WMW: 0.0018^**^ KS: < 2.2 × 10^−16^^***^	WMW: 3.233 × 10^−9^^***^ KS: 2.22 × 10^−7^^***^
6-month	WMW: 0.0593 KS: 0.0253^*^	WMW: 0.2956 KS: 0.3841	WMW: 0.0083^**^ KS: 0.0022^**^
7/11-month	–	–	WMW: 0.0063^**^ KS: 0.028^*^

^*a*^*p*-value significance is displayed as follows: ^*^, < 0.05; ^**^, < 0.01; ^***^, < 0.001.

### 3.4 Environmental factors

#### 3.4.1 Uranium in rocks and soils

**Figure 4:**
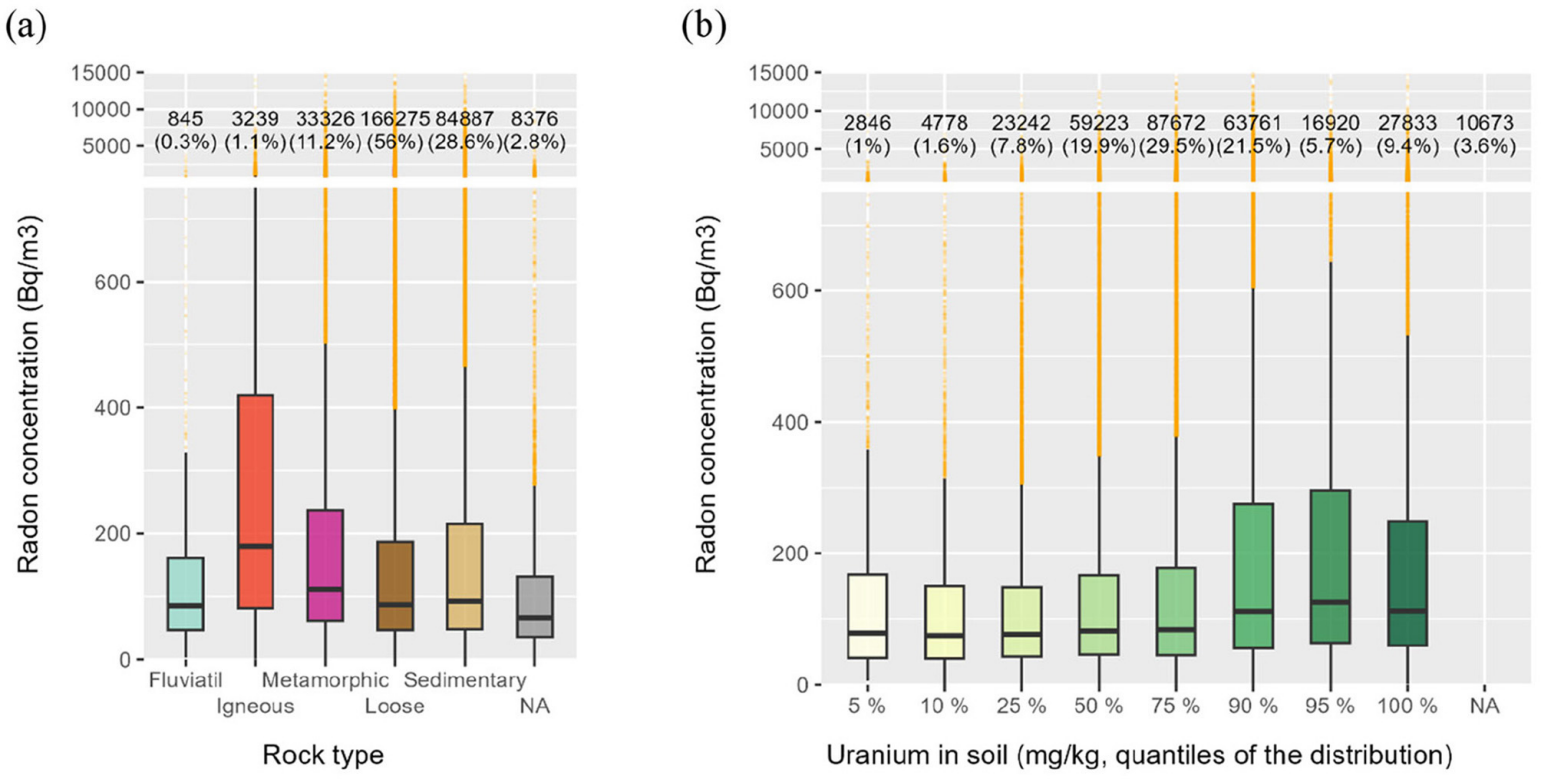
Radon concentrations distributed according to **(a)** rock type and **(b)** the uranium content in soils. The uranium content in soils is categorized according to the percentile of the distribution (in mg/kg) of all measurement carried out in Switzerland and is provided by the Geochemical Atlas of Switzerland (34, 35).

Radon concentrations have been extensively studied and are strongly associated with specific rock types, especially uranium-rich rocks (3, 9, 32, 33). Switzerland follows this characteristic, as shown in Figure 4. Figure 4a highlights elevated radon levels in regions with igneous rocks, which are widespread in the country. The radon concentration distribution over igneous rocks is significantly different from all other rock types, with Wilcoxon–Mann–Whitney test *p*-values below 2.2 × 10^−16^. Although statistically distinct (*p*-values below 2.2 × 10^−16^), radon concentrations over metamorphic, loose, and sedimentary rocks are relatively similar, with median values of 111, 87, and 93 Bq/m3, respectively. Figure 4b illustrates radon concentration distributions based on uranium content in soils. Overall, higher uranium concentrations in soil correspond to increased indoor radon levels. The three highest percentiles of uranium distribution (34, 35) exhibit statistically significant differences. Wilcoxon–Mann–Whitney test *p*-values were all below 2.2 × 10^−16^ when comparing these distributions with the 75th percentile. Figure 4 highlights the strong relationship between indoor radon levels and uranium presence in both rocks and soils.

### 3.4.2 Proximity to faults and seismic zones

**Figure 5:**
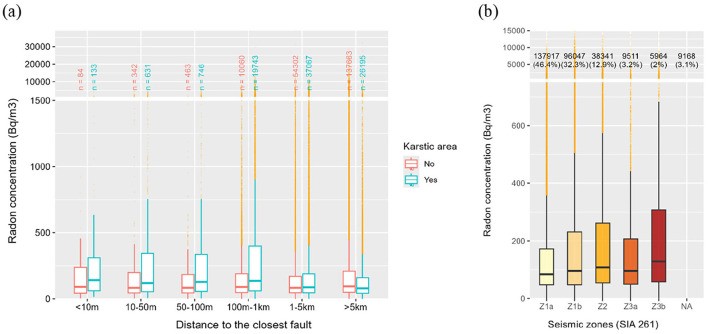
Boxplot distributions of indoor radon levels based on **(a)** the distance between the building and the nearest fault and **(b)** the building regulations required to meet seismic safety standards (SIA 261).

A second geological aspect influencing radon levels is its relationship with tectonic activity. These geological features can manifest as faults (Figure 5a) or seismic activity (Figure 5b). Figure 5a illustrates the relationship between indoor radon concentrations and proximity to the nearest fault, distinguishing between karstic and non-karstic environments. Radon levels are generally higher in buildings located within one kilometer of a fault, especially in karstic areas, a trend statistically confirmed by Wilcoxon-Mann-Whitney test *p*-values, all below 0.05. These results highlight the significant influence of karstic networks on indoor radon levels. Beyond 1 km from a fault, differences in radon concentrations remain minimal. From a seismic perspective, it is noteworthy that zone Z3b (Figure 5b), which has the highest seismic potential (36), also exhibits the highest overall radon concentrations.

### 3.4.3 Climatic norms

**Figure 6:**
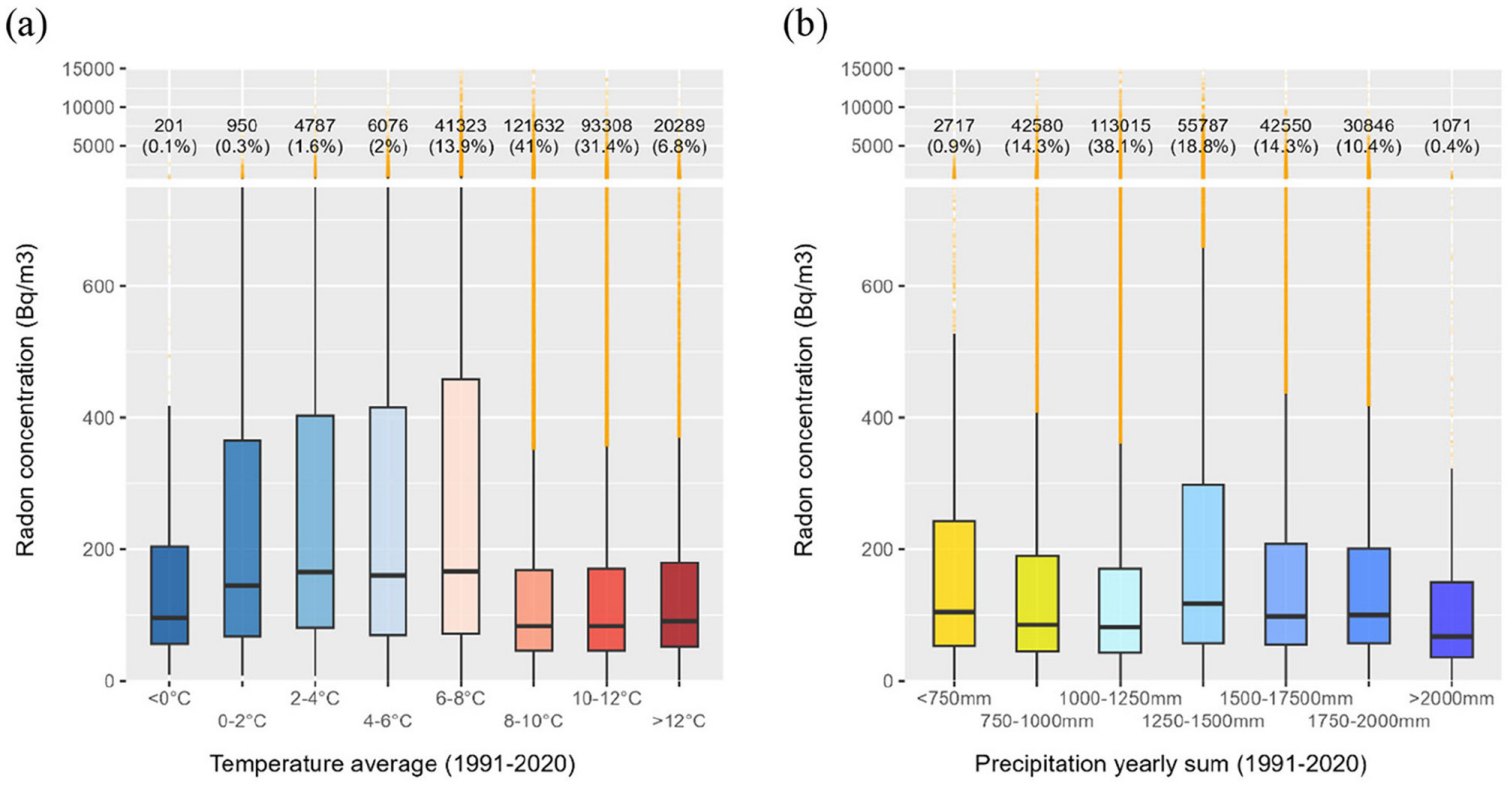
Boxplot distributions of indoor radon levels based on **(a)** average outdoor air temperature for the period 1991–2020 and **(b)** the average yearly sum of precipitation for the period 1991–2020.

Figures 6a, b show the distribution of indoor radon concentrations based on temperature and precipitation threshold norms for the period 1991–2020. Indoor radon levels tend to be lower in more temperate regions (average temperature > 8 °C) compared to colder areas (average temperature < 8 °C), as confirmed by a Wilcoxon–Mann–Whitney test (*p*-value < 2.2 × 10^−16^), indicating a statistically significant difference between the temperate and colder areas. Outdoor temperature is strongly influenced by geographic factors, including altitude, which affect occupant behavior, such as prolonged heating periods, that can lead to elevated indoor radon concentrations. Figure 6b depicts no significant trend between the average yearly sum of precipitation and indoor radon concentrations.

### 3.5 Building characteristics

**Figure 7:**
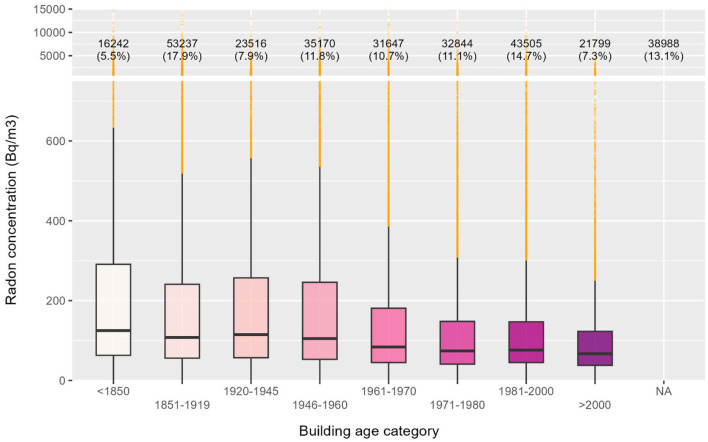
Radon concentrations distributed according to building age category applicable within the Swiss context.

Building age influences indoor radon levels due to changes in foundation integrity, ventilation, and construction practices. Indeed, older buildings often have more entry points, while newer ones may trap radon due to airtight designs (10, 26). Figure 7 illustrates radon levels across different construction periods available in Switzerland, each characterized by distinct building typologies (37). Newer buildings, regardless of airtight performance, generally exhibit lower radon concentrations. All distributions were statistically different, with Wilcoxon–Mann–Whitney test *p*-values below 0.05.

**Figure 8:**
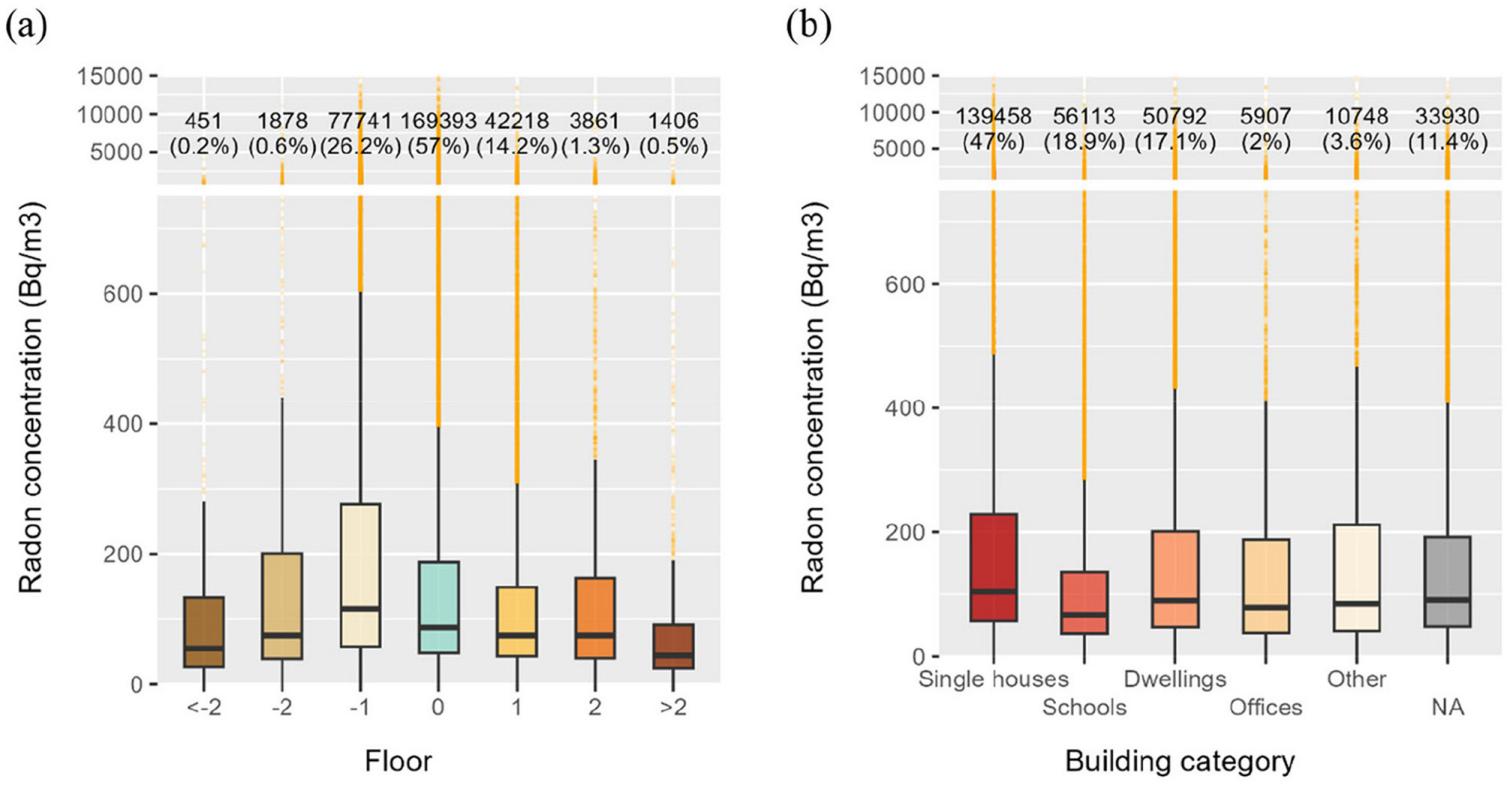
Radon concentrations distributed according to **(a)** the levels where measurements were carried and **(b)** the building category.

Figure 8a illustrates the distribution of radon levels in Switzerland according to building level. Overall, lower levels tend to exhibit higher radon concentrations. Notably, nearly one-quarter of the measurements conducted at level −1 exceed 300 Bq/m^3^. Conversely, at upper levels (>0), most measurements above 300 Bq/m^3^ are classified as outliers. It is important to note that measurements taken below the −1 floor are rare and tend to show lower radon levels than those in typical basements. Measurements from −2 floors and lower typically originate from unoccupied, well-ventilated spaces, such as garages or utility rooms, where radon accumulation is less likely due to increased air exchange. The Wilcoxon–Mann–Whitney test was used to assess statistical differences in radon concentration distributions across floor levels. All differences between floor levels distributions were statistically significant (*p* < 0.05), except for distributions between floor levels −2, 1, and 2, which were characterized by *p*-values above 0.05, thus depicting statistically similar distributions.

Figure 8b highlights similarities between the distributions for each building type, except in schools, where concentrations were lower. This may be explained by the fact that schools are well-ventilated, either naturally or mechanically, and generally have large interior volumes, which helps dilute radon concentrations. Despite apparent similarities, all differences between distributions were statistically significant (*p* < 0.05) based on the Wilcoxon–Mann–Whitney tests.

## 4 Discussion

Understanding indoor radon concentrations and their influencing factors is essential for improving public health strategies and risk mitigation. This paper presents the results of an in-depth analysis of the Swiss radon database, highlighting key insights into dataset completeness, radon distribution, geological influences, and building characteristics.

The analysis of the dataset revealed substantial disparities in data completeness across different variables. While some variables were consistently recorded, others were sparsely populated, limiting the depth of analysis. To enhance the utility of national radon databases for future research, it is crucial for relevant stakeholders to systematically enter complete information. Health authorities should consider automatically linking buildings in their database with data available in the federal register of buildings to ensure consistency among the databases. A more comprehensive dataset would allow for more precise assessments of radon exposure risks and support the development of targeted mitigation measures. Most of radon measurements, and by extension building investigated, conducted in Switzerland remain below the reference level of 300 Bq/m^3^. Approximately 17% of the measurements, representing 30,390 buildings, exceed this threshold, indicating localized areas of concern (38), although the whole country must be considered as a radon prone area (4). As a comparison, 15,000 and 3,000 measurements exceeded 300 and 1,000 Bq/m^3^ respectively in 2010. At the time, 12% of the building stock was expected to exceed the reference value of 300 Bq/m^3^ (39, 40). Since 2021, the FOPH has developed remediation deadlines recommendations based on radon levels (41). These results reveal that more than 30,000 buildings in Switzerland may be affected by these remediation deadline recommendations.

The duration of measurement is a determining factor in the assessment of indoor radon levels. Shorter measurement periods tend to yield higher observed radon concentrations. This raises the question of the annual representativeness of data obtained from shorter measurement durations (7, 15, 42–44). In the case of 3-month measurements conducted at different times of the year (i.e., across various seasons) no significant differences were observed between summer and winter. This finding is consistent with a recent study carried out in Switzerland by Rey et al. (42). It therefore raises questions of the current practice, which recognizes only 3-month measurements conducted during the heating period (October–March) as official. Before accepting 3-month measurements conducted throughout the year as equally official, it is important to carry out further research. At first, a climatic analysis of the length of the heating period over the overall territory of Switzerland may be helpful to assess the relevance of applying seasonal corrections factors. If needed, seasonal corrections factors could be evaluated for the present climate in Switzerland.

As a decay product of uranium, radon is strongly associated with uranium-rich geological formations. The results of this study confirm that uranium-bearing rocks, particularly igneous formations, and uranium-rich soils contribute to elevated indoor radon levels. These findings align with previous research conducted worldwide (9, 13, 32), reinforcing the well-established link between geology and radon distribution. In addition to uranium content, karstic networks, formed in limestone regions, have been identified as a significant contributor to radon variability. Although limestone itself contains relatively low uranium concentrations, karstic systems are known to facilitate radon transport (45). Faults within karstic networks may accelerate gas migration, increasing radon concentrations in overlying buildings (46–49). This study confirms that buildings situated above karstic formations are more prone to elevated radon levels. Finally, meteorological conditions have been identified as a strong influence of meteorological conditions on indoor radon levels in Switzerland (7, 13, 50) and abroad (43, 51). The present study confirms the inverse correlation between average temperature and indoor radon concentrations. However, when analyzing the average yearly sum of precipitation, no clear trends were identified, despite such relationships having been observed in previous studies (52).

Building age and floor level emerged as building key determinants of indoor radon concentrations. In general, older buildings and lower floor levels were more likely to exceed Switzerland's radon reference value. These findings are consistent with previous studies indicating that older construction techniques and materials may contribute to higher radon infiltration (13, 14, 53–59). Similarly, ground-level and basement spaces, being in direct contact with radon-emitting soils, exhibit a greater risk of elevated radon concentrations (14, 53, 55). However, it is important to note that some studies have highlighted the significant impact of energy retrofitting on indoor radon concentrations and, more broadly, on indoor air quality (IAQ). Indeed, IAQ has been deteriorated following energy retrofits in Switzerland (14) and internationally (53, 60–64). Finally, the analysis of indoor radon concentrations by building type did not reveal significant differences between categories, except for schools, where concentrations were lower. This finding complements previous literature reviews (54, 58, 59, 65). Other parameters, such as building foundations, were too inconsistent to be reliably analyzed. This is regrettable, as foundations have been shown to play a crucial role in indoor radon entry (14, 53, 55, 58).

### 4.1 Study limitations

The radon database is completed by trained professionals approved by FOPH. Despite their training and ongoing education, occasional errors may still be inadvertently introduced. However, the large size of the dataset helps minimize the impact of any potential errors. A second limitation lies in the variability introduced by the use of different instruments. However, in more than 75% of cases, alpha-track detectors were used.

## 5 Conclusion

Switzerland's radon database is one of the largest of its kind, providing valuable insights into indoor radon distribution. However, despite its extensive coverage, some variables remain only partially recorded. To maximize its usefulness for research and public health applications, it is essential for all stakeholders to ensure complete and accurate data entry when recording new measurements.

The analysis presented in this study highlights key factors influencing indoor radon concentrations. While high radon levels can occur across all geological regions, they are more prevalent in granitic areas and karstic networks. Additionally, proximity to faults increases the likelihood of elevated radon levels in buildings. As a result, structures built in karstic and highly fractured alpine regions should integrate the latest radon mitigation strategies. Furthermore, certain building characteristics, such as age and construction type, are strongly associated with indoor radon potential, emphasizing the need for targeted prevention measures.

Understanding the interplay between indoor radon, building characteristics, and environmental factors is crucial for future predictive modeling. This study demonstrates that some variables exert a greater influence on indoor radon concentrations than others, highlighting the importance of prioritizing these factors in future research and mitigation efforts.

After 40 years of radon measurements in Switzerland, the database now allows researchers to identify critical determinants of high indoor radon levels. These findings must be effectively communicated to relevant stakeholders, including authorities, architects, engineers, and construction professionals. Finally, radon research must be better coordinated at the European level to provide a more comprehensive overview of the issue. This paper calls for greater consistency in data collection (same variables for instance), at least among neighboring countries. This would ensure long-term cross-country comparisons and enable the assessment of the effectiveness of different public health policies. Ultimately, these joint measures would help reduce the population's exposure to radon.

## Data Availability

The data analyzed in this study is subject to the following licenses/restrictions: Radon dataset is not publicly available. Data belongs to Switzerland Federal Office of Public Health and the use is restricted. Requests to access these datasets should be directed to FOPH website (https://www.bag.admin.ch/bag/en/home/gesund-leben/umwelt-und-gesundheit/strahlung-radioaktivitaet-schall/radon.html).

## Supplementary material

Supplementary Figure S1 Radon concentrations distributed according to measurement length (1-month: < 59 days; 2-months: 59–88 days; 3-months: 89–118 days; 4/5-months: 119–179 days; 6-months: 180–210; 7/11-months: 211–364; 12-months: >364 days).

Supplementary Table S1 Full list of variable completion scores with their relevant description.
