# Supplementary figures and images for: Swiss national radon database: impact of building and environmental factors
Source: Front Public Health. 2025 Aug 22;13:1625922. doi: 10.3389/fpubh.2025.1625922 (PMC12411543; doi:10.3389/fpubh.2025.1625922)

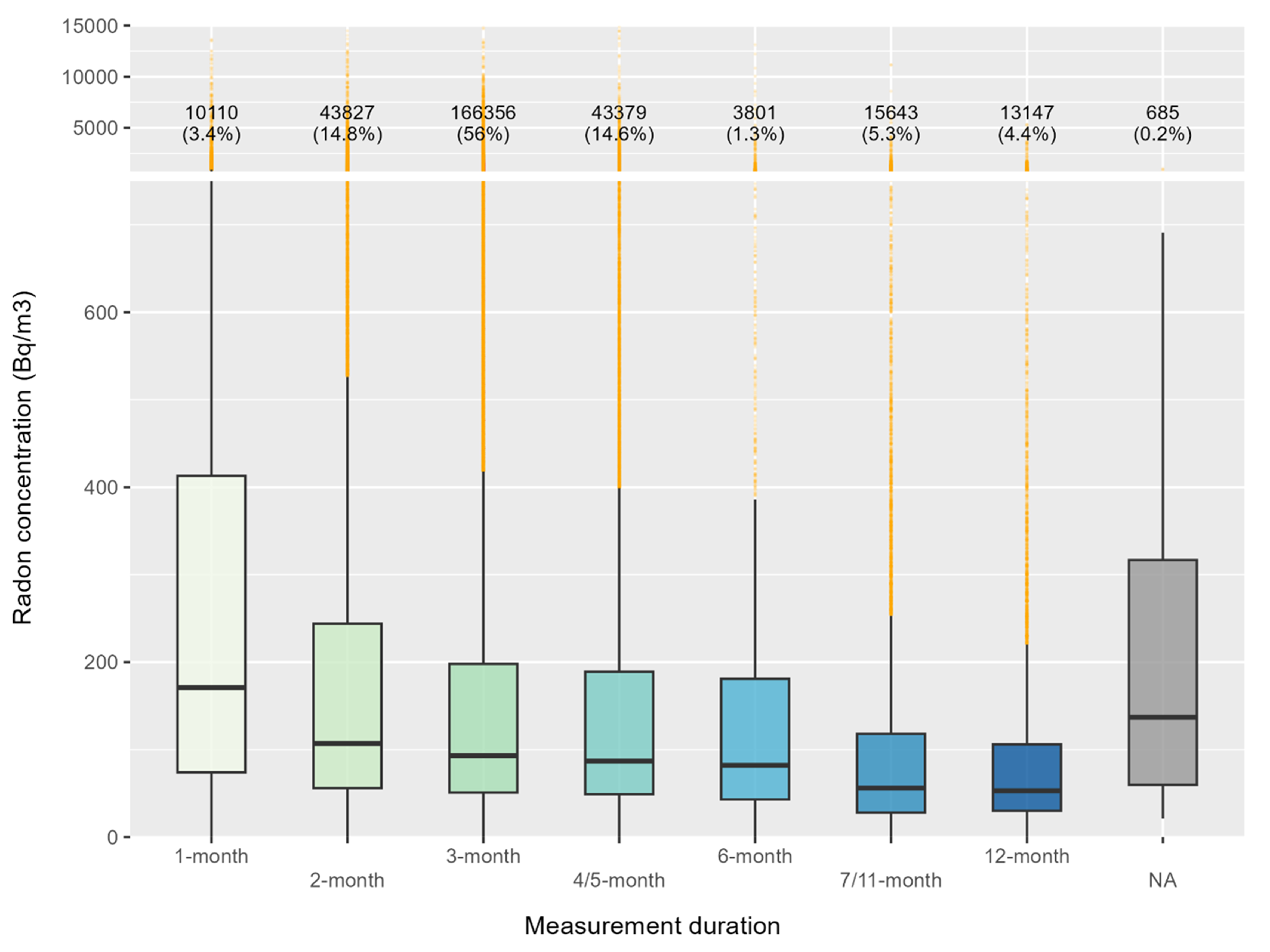

Supplement: Supplementary Figure S1 — Radon concentrations distributed according to measurement length (1-month: < 59 days; 2-months: 59–88 days; 3-months: 89–118 days; 4/5-months: 119–179 days; 6-months: 180–210; 7/11-months: 211–364; 12-months: >364 days). [file Image_1.jpeg]
