# Supplementary material for: Swiss national radon database: impact of building and environmental factors
Source: Front Public Health. 2025 Aug 22;13:1625922. doi: 10.3389/fpubh.2025.1625922 (PMC12411543; doi:10.3389/fpubh.2025.1625922)
Supplement: Supplementary Table S1 — Full list of variable completion scores with their relevant description. [file Table_1.docx]

**Table S1**: Full list of variable completion scores with their relevant description.

|  | **Variable** | **% of completion** | **Description** |
| --- | --- | --- | --- |
| 1 | Canton | 100 | Swiss canton |
| 2 | Building ID | 100 | Identification number of the building |
| 3 | Address | 100 | Complete postal address of the building |
| 4 | Postal number | 100 | Swiss postal number |
| 5 | Municipality ID | 100 | Identification number of the municipality |
| 6 | Municipality | 100 | Swiss municipality |
| 7 | Building category | 100 | Category of the building |
| 8 | Geo coordinate source | 100 | Geographical coordinate source |
| 9 | ID Measurement | 100 | Identification number of the measurement |
| 10 | Measurement protocol | 100 | Protocol that has been used for the measurement |
| 11 | Measure type | 100 | Type of measurements |
| 12 | Measurement instrument ID | 100 | Identification number of the measurement instrument |
| 13 | Measurement instrument type | 100 | Type of measurements instrument |
| 14 | Space ID | 100 | Identification number of space |
| 15 | Space Type | 100 | Type of space |
| 16 | Occupancy | 100 | Occupancy of the space |
| 17 | Floor | 100 | Floor where the measurement took place |
| 18 | Mitigation deadline (if applicable) | 100 | Mitigation deadline relative to measurement results (if applicable) |
| 19 | Detection method | 100 | Detection method |
| 20 | Floor category | 100 | Category of floor |
| 21 | Location | 99.99 | Name of the location (village) if different from municipality |
| 22 | Measurement beginning | 99.97 | Date of measurement start |
| 23 | Measurement end | 99.77 | Date of measurement end |
| 24 | Radon concentration | 99.54 | Radon concentration |
| 25 | Radon exposition | 98.11 | Radon exposition |
| 26 | Occupancy (hour/year) | 97.92 | Occupancy |
| 27 | Geo. Coordinate LV03 – North | 97.84 | Geographical coordinates LV03 – North |
| 28 | Geo. Coordinate LV03 – East | 97.84 | Geographical coordinates LV03 – East |
| 29 | Radon uncertainty | 91.07 | Radon measurement uncertainty |
| 30 | Building year | 90.63 | Year when the building has been built |
| 31 | Building category | 88.57 | Building category |
| 32 | Building foundations | 82.37 | Foundations building |
| 33 | Geo. Coordinate LV95 – North | 72.30 | Geographical coordinates LV95 – North |
| 34 | Geo. Coordinate LV95 – East | 72.30 | Geographical coordinates LV95 – East |
| 35 | No parcelle | 71.99 | Number of the parcel (cadaster) |
| 36 | EGID | 71.86 | Building federal dentification number |
| 37 | Address (old) | 43.03 | Adress of the building (old category) |
| 38 | Building number of floors | 24.16 | Total number of floors of the building |
| 39 | Slope | 18.02 | Slope of the parcel |
| 40 | Number of underground floors | 17.77 | Total number of floors underground of the building |
| 41 | Mechanical ventilation | 17.37 | Presence of a mechanical ventilation |
| 42 | Foundation structure | 16.77 | Structure of the foundation |
| 43 | Equilibrium factor  (workplace only) | 0.12 | Equilibrium factor only for workplaces. |
| 44 | Effective dose  (workplace only) | 0.12 | Effective dose calculated only for workplaces. |
